# A comparative study on the availability of modern contraceptives in public and private health facilities in a peri-urban community in Ghana

**DOI:** 10.1186/s12978-015-0058-z

**Published:** 2015-08-08

**Authors:** Kwame K. Adjei, Amos K Laar, Clement T. Narh, Martha A. Abdulai, Sam Newton, Seth Owusu-Agyei, Sam Adjei

**Affiliations:** Kintampo Health Research Centre, Ghana Health Service, Kintampo, Ghana; Department of Population, Family and Reproductive Health, University of Ghana, Legon Accra, Ghana; University of Health and Allied Sciences, Ho, Ghana; Centre for Health and Social Services, Accra, Ghana

**Keywords:** Availability, Contraceptives, Factors, Health facilities

## Abstract

**Background:**

Sub-Saharan Africa reports low use of family planning methods and high unmet need. Availability of these methods is one of the major barriers to contraceptive use in the region. This study determined the availability of modern contraceptives and perceived factors affecting this in health facilities in the Ga East municipality of Ghana.

**Methods:**

This was a cross-sectional study involving quantitative and qualitative techniques. Data was obtained from 51 randomly selected health facilities using a checklist. Relationships between certain attributes of the facilities and availability of each category of contraceptive identified was tested using univariate and and multiple logistic regression techniques. The qualitative data was obtained by conducting in-depth interviews with the managers of the facilities and then analysed according to emerging themes.

**Results:**

The study gave an indication that there was a low availability of long acting reversible contraceptives (LARC) such as implants (14 %) and IUDs (14 %) in the health facilities. Male condoms (78 %) and combined oral contraceptives (82 %) were the most available At the bivariate level, emergency contraceptives were less likely to be found in public health facilities (OR = 0.11, *p* = 0.05). Facility managers cited ‘profit’ and ‘preference’ as some of the reasons for availability of their contraceptives.

**Conclusion:**

Availability of modern contraceptives differ according to the type and brand of contraceptive. There is however a low availability of LARC methods in all the health facilities. Factors such as ‘profit’ accounted for the low availability of this method.

## Background

Various studies conducted show that unintended pregnancies and related consequences such as abortion are as a result of reasons such as low availability and non-use of contraceptives [1–3]. Sub-Saharan African (SSA) countries are the most affected as they have persistent high rates of unmet need for family planning and low contraceptive use [4–6]. Countries such as Tanzania and Nigeria record high unmet needs and low contraceptive use [7, 8].

In Ghana, the modern contraceptive prevalence as reported by the Ghana Health Demographic Survey [9] was as low as 17 % with unmet need of family planning for women between 15 and 49 years at 35 % and total fertility rate (TFR) at 4.0. Ghana was one of the first African countries to adopt and formulate a National Population Policy in 1969 which was revised in 1994. The revised national policy states the aims of the family planning component in the reproductive health policy and these include: provision of information to individuals as well as making available a full range of safe and effective contraceptive methods [10].

The Ghana Demographic and Health Survey [9] has awareness of modern contraceptives amongst women of reproductive age at 98 %. The use of these methods varied according to the commodity, with injectables and pills as the most preferred methods. Information on the availability of these family planning commodities in health facilities in Ghana is however limited [9]. The importance of making available family planning commodities in order to improve contraceptive use and family planning coverage in Ghana is emphasized in a recently released Ghana Millennium Development Goals Acceleration Frame work and Country Action Plan [11].

The Ghana Millennium Development Goals framework states “making available family planning commodities in health facilities” one of the major bottlenecks that needs to be addressed in order to improve maternal health in Ghana [11]. Making available family planning commodities is also linked with improved accessibility to family planning, increased family planning coverage as well as acceptability of these methods; all these lead to improved maternal and neonatal health [11].

Ghana is known for operating a pluralistic health system which is guided by policies and legislations such as the Ghana Health Service and Teaching Act 525 amongst others [12]. Health care in the country is obtained primarily from two main providers: the private and the public. Private providers in Ghana usually include ‘not for profit’ organizations and private practitioners who operate private health facilities such as clinics, pharmacies and licensed chemical shops [12, 13]. Private health facilities in Ghana such as pharmacies obtain their medical supplies and other required logistics primarily from registered private organizations such as pharmaceutical companies [13]. Public providers on the other hand are associated with government institutions such as public hospitals, health centres and the community-based health planning and services (CHPS) [13]. Public health facilities in Ghana obtain their medical supplies and other required logistics primarily from the Ghana medical stores [14, 15]. Health care from private providers is perceived to be better than public health care [13]. It is estimated that more than fifty percent (50 %) of Ghanaians obtain their health care including contraceptives from private providers [15].

This study sought to assess the availability of modern contraceptive methods in public and private health facilities and factors affecting this as perceived by facility managers in the Ga East municipality. A comparative analytical approach was used to measure this outcome.

## Methods

### Study design

A cross-sectional design was used allowing for a comparative analytical approach. A mixed-methods approach involving quantitative and qualitative data collection techniques was used.

### Study area

Ghana is divided into districts, municipalities and metropolis Data for the study was collected in the Ga East municipality, a predominantly peri-urban community located in the Greater Accra region of Ghana [16]. The rapidly developing population makes it ideal to study. The municipality is made up of four sub-municipalities with a total population of 320,853 and over 100 health facilities ranging from hospitals and clinics to pharmacies and licensed chemical shops [16].

### Sampling methods and data collection

The health facilities in the municipality served as the sampling frame for the study. For the purposes of the comparative analytical approach, the health facilities were first divided into two broad arms: private and public health institutions. These two broad arms were made up of four major types of health facilities depending on the level of care each facility provided. These four types of health facilities were the only available health facilities where clients could obtain family planning commodities in the district. The four types were: hospitals, clinics//maternity homes/health care centres, pharmacies and licensed chemical shops. A sample of 51 facilities from an estimated 150 health facilities were used. These were made up of 43 private and 8 public health facilities.

The municipality had only eight public health facilities where family planning services were rendered and all were used for this study. For the private institutions, a total of three different lists covering all the private health facilities in the municipality was employed to select the final sample. The first list comprising all maternity homes/clinics/ health centres and hospitals in the municipality where family planning services were provided was obtained from the municipality’s health directorate in Abokobi, the capital of the municipality. Health facilities where family planning services were not provided were excluded from the list.

The municipality’s health directorate could not provide a clear cut list for pharmacies and chemical shops. However, they were instrumental in the acquisition of two representative lists covering all pharmacies and chemical shops. These were obtained from the government facilities in the four sub-municipalities under the municipality.

The three different lists showed that pharmacies provided the most number of private health facilities with hospitals providing the least. The forty three (43) private facilities used were distributed as follows: pharmacies 20 (45 %), chemical shops 13 (35 %), clinics/polyclinics/health centres/maternity homes 7 (15 %) and hospitals 3 (5 %). This was in accordance with the proportion each category of health facility contributed.

Simple random sampling was employed to select from each of the three lists the required number of facility under each category. With the help of the community health nurses in the respective sub-municipalities, each of the randomly selected facilities under the various categories was located and the availability of modern contraceptives in that facility determined using a checklist. We adopted and adapted a previously used checklist in a related study [17]. The current study’s checklist, which was pre-tested in a few selected health facilities outside the study area, had four main sections with corresponding questions ranging from the age and sex of the respondent to the name and location of the health facility. The checklist defined availability of a contraceptive as follows:

For a contraceptive to be available, the facility manager first of all indicated that the facility had that contraceptive in stock as in Bowen *et al.* [17]. Additionally, the current study impressed upon facility managers to provide evidence; and this was observed by the trained data collection personnel. Finally, for each type of contraceptive, the trained personnel ensured that the quantity in stock met the minimum contraceptive stock level for that facility.

The facility manager present in each selected facility was also interviewed by a trained personnel and the interview recorded. The in-depth interview guide used by the trained personnel also had four main sections covering key indicators such as factors affecting availability and cost of modern contraceptives in the facility. A total of 51 in-depth interviews (IDIS) were conducted amongst the facility managers.

### Power calculations

A descriptive study by Bowen *et al.* [17] provided information on the availability of male condoms and IUDs in the municipality and this was used in the calculating the sample size. The availability of male condoms in the government health facilities was 71 % whilst that of the private health facilities was 25 %. Given the lack of data on the other modern contraceptives, this was used as a proxy. Using a 95 % confidence level and a statistical power of 80 %, the minimum sample size of 48 was obtained [18] as illustrated below. At the end of the study however, 51 facilities participated.

***Sample size calculations (illustration)***

**Table:** 

Key outcome of interest	Expected frequency of outcome in public health facility	Expected frequency of outcome in private health farcicality	Confidence level	Statistical power	Minimum Sample size
Availability of modern contraceptives (male condom used as a proxy)	71 %	25 %	95 %	80 %	48

### Data analysis and management

A comparative assessment of the two groups, private and public health facilities was done after data entry and cleaning. We used univariate analysis to generate descriptive tabulations for key variables. Statistical tools such as frequency distribution tables and cross tabulation were used to compare the availability of modern contraceptives in public and primary health facilities. Analysis looking at the associations between certain attributes of the facilities (type, place, and average number of clients per day) and the main outcome variable, availability of each category of contraceptive identified, was tested using logistic regression. Associations were assessed using multiple logistic regression techniques, where odds ratios (ORs) and their 95 % confidence intervals (CI) were computed. We employed a standard logistic regression modelling in SPSS (the “Enter” method) in our analysis. With this method, all the variables previously reported to be associated with the outcome variable or found to be associated with the outcome during the bivariate analysis were entered and a full model generated in a single step. A key attribute of each model (R^2^) are included in Table 2. *P* value <0.05 was used to denote statistical significance. All analyses were performed using IBM SPSS Statistics for Windows, Version 20.0.

The key outcome variables assessed in this study was fertility intentions/childbearing desires (the desire to a child or children in the near future), awareness and use of contraceptives (being aware of and usage of various contraceptive options available to HIV-positive women).

For the qualitative aspect, the running notes from the in-depth-interviews were written into expanded notes. The audio recordings were also transcribed and read several times to clean the data. The information collected was listed in Microsoft word 2010 and then categorized manually into emerging themes. The themes were not determined prior to the start of the study.

### Ethical approval and informed consent

Ethical clearance was obtained from the Ghana Health ervice Ethical Review Committee of the Research and Development Division of the Ghana Health Service. In addition to the ethical clearance, permission was also sought from the Municipal director of the Ga East municipal health directorate, as well as the facility managers of the various private and public health facilities.

Informed consent was sought from the facility managers of the various health facilities and it included: the purpose of the study, the risks and benefits, privacy and confidentiality as well as conflict of interest.

## Results

### Background characteristics

The health facilities in Ga East where contraceptives are sold were identified and categorized into two broad groups: public and private health facilities. The public health facilities were eight (8) in number and they included one (1) hospital, seven (7) health centres/clinics/polyclinics. The private health facilities used were 43 in number and these were: 13 chemical shops, 20 pharmacies, 3 hospitals and 7 health centres/maternity homes/clinics.

### Range and brand of contraceptives

Eleven different categories of contraceptives in the municipality were identified and distributed in the health facilities as follows: combined oral contraceptives (82 %), progestin only pills (18 %), emergency contraceptives (49 %), progestin only injectable (35 %), monthly injectable (22 %), spermicides (39 %), female condoms (4 %), male condoms (78 %), copper intra-uterine devices (14 %), implants (14 %) and the ovulation predictor (2.3 %). The common brands under each category of contraceptives available in the facilities include: *Microgynon, Secure, Depo Provera, Postinor 2, Champion condom* and *Norigynon*. These brands were available in more than one of the health facilities visited (Table 1). Male condoms had the most brands available (37) but they were specific to certain facilities. The long acting reversible contraceptives (LARC) such as implants and IUDs had as well as the female condoms had only one brand available (Table 1).

**Table 1:** Brand of contraceptives in private and public health facilities

Type of contraceptive	Private health facilities				Public health facilities	
	Hospital	Pharmacy	LC S	Clinic/H.C/M. H	Hospital Clinic/H.C/M.H/P. C	
Combined oral contraceptives		Frequency (*n* = 43)				Frequency (*n* = 8)
Microgynon	–	1	–	2	1	5
Secure	1	19	12	1	–	–
Emergency contraceptive						
WhitePostinor2	–	11	8	–	–	1
GreenPostinor2	–	8	1	–	–	–
Long term contraceptives						
Jadelle	1	–	–	3	–	3
Copper IUD	2	–	–	3	–	3
Injectables						
Depo Provera	2	1	–	7	1	7
Norigynon	1	–	–	2	1	7
Condoms						
Female condoms	–	–	–	1	–	1
Be Safe	–	9	5	1	1	4
Protector plus	–	14	7	1	–	–
Lubricated	1	5	2	–	–	2

### Availability of contraceptives in public and private health facilities

**Fig. 1:**
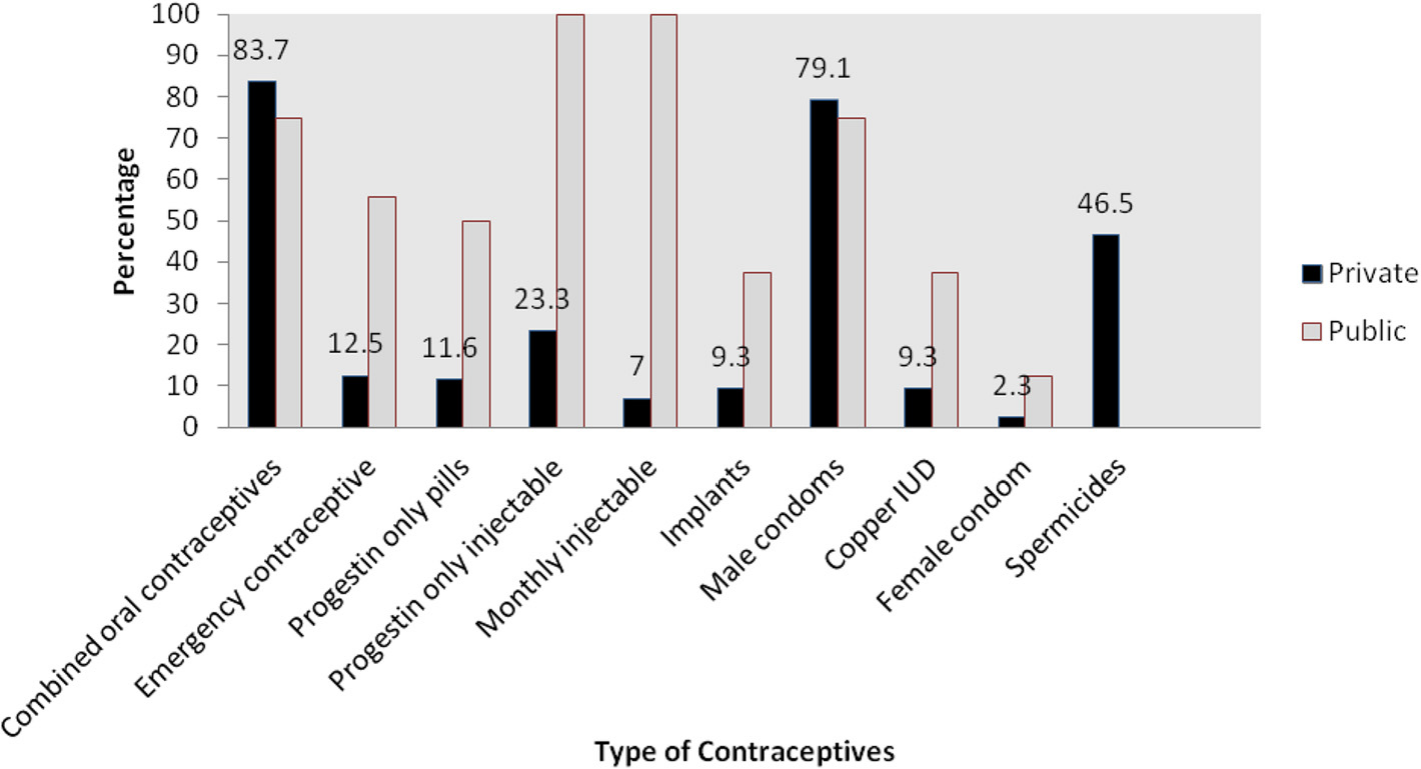
Contraceptives available in private and public health facilities

The most available modern contraceptive in the health facilities was the combined oral contraceptives. Eighty four (84 %) of private facilities had oral contraceptives compared to (75 %) of public facilities (Fig. 1). The public hospital available in the municipality (100 %) and (71 %) of the public clinic/maternity had these oral contraceptives. Comparatively, all the pharmacies in the municipality (100 %) and 92 % of the LCS also had these oral contraceptives. Only 33 % of the private hospitals and 43 % of the private clinics/maternity in the municipality had these oral contraceptives. There was no statistically significant relationship between availability of combined oral contraceptives and the type of health facility (private/public) (Table 2).

The male condom was another of the modern contraceptives that was highly available in the Ga East municipality. Seventy nine (79 %) of private facilities had male condoms compared to (75 %) of public facilities (Fig. 1, Table 1). The male condoms were mostly available in pharmacies (95 %) and LCS (92 %). Only 33 % of the private hospitals and 28 % of private clinics/maternity in the municipality had these male condoms. Comparatively, (71 %) of the public clinic/maternity homes and the public hospital (100 %) had the male condoms. From the multiple regression analysis, there was no association between availability of male condoms and type of health facility (private /public).

The perceived reasons for the high availability of condoms in pharmacies and LCS was summed up by one of the facility managers as follows:

*“People are learning and they want to protect themselves so most of these men go for condoms” (chemical shop assistant).*

All (100 %) public health facilities (both hospitals and clinics/maternity homes) had both the 3-month injectable and the monthly injectable available. The 3-months injectables was also available in all (100 %) of private clinic/maternity and 75 % of private hospitals. None of the LCS visited had the 3-months injectables whilst (5 %) of pharmacies had them available. The monthly injectables was not available in any of the LCS and pharmacies whilst 28 % of clinics/maternity homes and 33 % private hospitals had them in stock. The facility managers at the chemical shops and pharmacies offered reasonable explanations for not having injectables.

*“We do not give injections here. When clients come we tell them to go to the hospital or clinic. Those are the places where injections are given.” (pharmacy assistant)*

*“The injectables belong to the hospitals and clinics. We refer anyone who comes asking for some to those places.” (chemical assistant)*

The emergency contraceptives was available primarily in private health facilities such as pharmacies (75 %) and LCS (69 %). At the bivariate level, emergency contraceptives were less likely to be found in public health facilities (OR = 0.11, *p* = 0.05) (Table 2).

**Table 2:** Comparison of availability of modern contraceptives in public and private health facilities: Univariable and Multiple Logistic Regression

Contraceptive type available	OR^1^ [95 % CI]	OR^2^ [95 % CI]
Combined oral contraceptive		
Private	1	
Public	0.58 [0.10–3.50]	0.63 [0.33–11.84]
Progestin only		
Private	1	
Public	7.60* [1.43–40.39]	0.30 [0.02–4.71]
Male condoms		
Private	1	
Public	0.80 [0.14–4.62]	0.30 [0.01–7.46]
Implants		
Private	1	
Public	5.85* [1.01–34.10]	3.24 [0.11–93.07]
Copper IUD		
Private	1	
Public	5.85* [1.01–34.10]	3.24 [0.11–93.07]

For the multiple regression the following categories of contraceptives: the combined oral contraceptives, the male condoms, the implants and the copper IUD contained three predictor variables namely, the type of facility (eg LCS, pharmacy) under each health institution, the last time the facility was stocked with contraceptives and the average number of clients. The progestin only contraceptive contained two predictor variables namely the type of facility (eg LCS, pharmacy) under each health institution and the average number of clients per day

*OR* ^*1*^ Odds Ratio for univariable logistic regression, *OR* ^*2*^ Odds Ratio for multiple logistic regression analysis, *CI* ^*1*^ &*CI* ^*2*^ 95 % Confidence Interval for OR^1^ & OR^2^

*P value significant at 0.05

The increase use of emergency contraceptives in private health facilities such as LCS and pharmacies was summed up by one of the facility mangers as follows:

*“Most of the clients like the emergency contraceptives. I know someone who takes it virtually every day. She claims she can enjoy sex with her boyfriend and still prevent pregnancy.” (pharmacy assistant)*

One of the facility managers at the public health facilities also had this to say about the emergency contraceptives:

*“The clients abuse them when we give it to them. In order not for them to abuse it we do not sell the emergency contraceptives.” (public health nurse)*

The Jadelle implants and the copper IUDs were the long-acting reversible contraceptives (LARC) identified in both private and public health facilities. There were no LARC methods available in pharmacies and LCS. The implant was not available in the public hospital compared with 33 % of the private hospitals. Forty three percent (43 %) of both private and public clinics/maternity homes had implants. The copper IUD was available in twenty eight (28 %) of the private clinics/maternity homes as compared with 43 % public clinics/maternity homes. There was no statistically significant relationship between the availability of implants and copper IUDs and type of health facility (public/private). One of the main reasons for the low availability of LARC methods in both public and private health facilities particularly the hospitals and clinics/maternity was summarized by a facility manager as follows;

*“No one in this unit has been trained to do insertion of the IUDs or implants. My immediate boss will soon undergo the training but till then we do not sell the implants or IUDs.” (public health nurse)*

### Facility managers’ perspectives on factors affecting the availability of contraceptives in public and private health as perceived by facility managers

This study sought to determine why these facilities sold the particular type of contraceptives they had in stock. The major factors or themes that emerged from these discussions were ‘policies’, ‘training’, ‘competition’, ‘preference’ ‘demand’, ‘profit’ and ‘infrastructure’. Some of the facility managers talked extensively about these factors.

- **Demand-** “*The demand of the people determines what we have. We sell what people ask for. Some of these contraceptives do not sell fast and if you stock them they might expire.”(pharmacy assistant)*
- **Preference-***“The females themselves do not like the female condoms. They say it is difficult and unpleasant to use them and they prefer them as bangles. I only have a sample just for display.”(public health nurse)*
- **Policy-***“The pharmacy council does not allow us to sell certain drugs so we cannot sell injections” (chemical shop owner)*
- **Infrastructure-***“We do not have a place specifically for family planning. The maternity ward also doubles as the family planning unit. Plans are underway to put in place a family planning unit” (medical assistant)*

## Discussion

In the Ga East municipality of Ghana where this survey was carried out, eleven categories of modern contraceptive methods were identified ranging from combined oral contraceptives to spermicides.

For the private health facilities such as pharmacies and LCS, the combined oral contraceptives, the emergency contraceptives and the male condoms were the most available contraceptives compared with other modern contraceptives such as injectables and LARCs. The most available modern contraceptive in the public health facilities was the injectables and it was the only method available in all the public hospitals and clinics (100 %) in the municipality. Contrary to expectations, one private pharmacy also had available the injectables. None of the LCS had injectables available.

Hong *et al.* [19] lists the most available modern methods of contraceptives in Ghana as the pills, the injectables and the male condoms.

Findings from our study however indicates that the injectables might be available only to women who patronize public health facilities but not to those who patronize private health facilities like LCS and pharmacies. From the discussions with the facility managers, it was clear that the injectables were not available because of policies which do not allow the sale of these modern contraceptives in pharmacies and LCS.

In Ghana, LCS are on the increase and evidence shows that more women are patronizing these services [20]. Lebetkin *et al.* [20] recently in their study have shown that it is possible to equip these facilities to effectively administer these injectables. It would be great if policies surrounding the sale of these injectables are therefore re-looked at to enable the sale of these injectables at LCS and pharmacies. Safer and easier to handle injectables such as ‘sayana press’ which has also been shown to work in some selected countries can also be introduced to these facilities to increase the availability of injectables to women who patronize LCS and pharmacies [21].

The availability of long acting reversible contraceptives (LARC) methods such as the implants and the copper IUDs in this study was low with only 7 out of the 51 health facilities (14 %) having them in stock. None of chemical shops and pharmacies sampled had these LARC methods. The levenogestrel IUD was also not available in any of the health facilities surveyed. The facility managers in the LCS and pharmacies attributed this to policies on the sale of these LARC whilst those in the public hospitals and clinics attributed this to the lack of trained personnel on the insertion of these methods.

This observation highlights a growing challenge observed by Aryeetey *et al.* [6] in which it was made apparent that awareness of LARC methods in the Ga East municipality was low. It also adds to the growing perception that this is one of the key barriers that need to be addressed in order to increase the utilization of contraceptives in Ghana and in particular public health facilities, is the health service barrier [6, 15]. It also underlines the problem of inadequately trained staff in insertion of these methods since these methods can only be inserted in facilities with trained providers [15]. Findings from this study also suggest the utility of motivating an increase the number of private hospitals and clinics/ maternity homes as well as public hospitals and clinics/maternity health centres which offer family planning in the municipality if there is to be an increase in availability of LARC methods since these methods require surgical procedures that can be carried out only in these types of facilities. The introduction of the CHPs concept in this area and the probable setting up of CHPs compounds in the municipality is a step in the right direction.

Finally, this present study also demonstrated the different brands of contraceptives under each category of contraceptive identified. *Secure* was discovered as the most available brand of contraceptive pills particularly in private health facilities. This is in accordance with the Ghana Demographic and Health Survey [9] which reports that *Secure* is the most popular brand of contraceptive pills in Ghana. Other contraceptive pills identified during this study include, *Microgynon*, *Blue, Yasmin, Primolut-n, Overette, Microlut* and *Micronor*. The Ghana Demographic and Health Survey [9] confirms the availability of these brands in Ghana in addition to other brands such as *Lo-femenal, My pearl, N/M, OC, Duofem, Oral, Skill* and *Hot* which were not identified in the health facilities sampled for this study. A limitation of our study which was the small number of public health facilities available and hence a small sample of facilities were used could account for this. Nevertheless, the absence of other brands of contraceptive pills in this study as compared to the Ghana Demographic and Health Survey [9] could be an indication of the decline in the options available to users of this method particularly in this municipality and this could cause a subsequent decline in its use.

## Conclusions

This comparative study on the availability of modern contraceptives in both private and public health facilities has brought to light the low availability of some contraceptives particularly the female condom and LARC methods such as implants and IUDs in these facilities.

This study has also revealed the high availability of methods such as emergency contraceptives and spermicides in private institutions as compared with public ones. Methods such as the injectables are more available in public health facilities compared with private ones.

## Recommendations

We recommend that policies regarding the sale of injectables in LCS and pharmacies be re-looked at to increase availability and possible use of these methods. Increasing the number of trained personnel in LARC methods in both private and public hospitals as well as clinic/maternity homes may positively affect their availability in the currently authorized outlets.
